# Unveiling the Therapeutic Mechanisms of Chinese Herbs in Heart Failure: Integrating Network Pharmacology, Molecular Docking, and Simulation Analysis

**DOI:** 10.3390/ph18111648

**Published:** 2025-10-31

**Authors:** Basharat Ahmad, Cai-Yi Ma, Grace-Mercure Bakanina Kissanga, Sebu Aboma Temesgen, Huma Fida, Hao Lin, Cheng-Bing Huang

**Affiliations:** 1 School of Life Science and Technology, Center for Informational Biology, University of Electronic Science and Technology of China, Chengdu 610054, China; basharatahmad3674@gmail.com (B.A.);; 2 School of Computer Science and Technology, Aba Teachers University, Aba 623002, China

**Keywords:** heart failure, traditional Chinese medicines system pharmacology, protein–protein interaction, molecular dynamics simulation

## Abstract

**Background:** Heart failure (HF) is a leading cause of mortality and represents the final stage of various cardiovascular disorders. Although traditional Chinese herbs have been extensively applied in HF treatment and their clinical efficacy has been investigated, the underlying molecular mechanisms remain insufficiently understood. To address this gap, systematic approaches are required to elucidate the therapeutic basis of herbal interventions. **Methods:** In this study, we systematically analyzed the bioactive compounds from seven traditional Chinese herbs, *Baiguo* (*Ginkgo biloba*), *Chishao* (*Radix Paeoniae Rubra*), *Biba* (*Piper longum*), *Aidicha* (*Ilex latifolia*), *Bajiaolian* (*Dysosma* spp.), *Beiwuweizi* (*Schisandra chinensis*), and *Baiqucai* (*Sedum sarmentosum*) and explored their potential mechanisms in HF by integrating network pharmacology, molecular docking, and molecular dynamics simulations. **Result:** We identified key targets and pathways implicated in HF pathogenesis and herbal interventions. A total of 63 active compounds were found to regulate 1947 genes. Through integrative analysis of the GSE57338 heart failure dataset from the GEO database, we further identified 265 intersecting targets shared between herb-associated genes and HF-related genes, highlighting their potential involvement in HF progression. Network analysis prioritized three hub proteins, STAT3, SRC, and TP53, which were subsequently subjected to molecular docking with the top bioactive compounds (quercetin, kaempferol, and epigallocatechin-3-gallate). Docking studies revealed strong binding affinities, and molecular dynamics (MD) simulations further validated the stability of these protein compound interactions. **Conclusions:** This study elucidates key bioactive components and targets involved in HF treatment, with kaempferol and epigallocatechin-3-gallate emerging as promising therapeutic candidates. These results provide a foundation for future experimental validation and the development of targeted HF therapies derived from traditional Chinese medicine.

## 1. Introduction

Heart failure (HF) is a complex clinical syndrome in which impaired cardiac function results in inadequate blood circulation, failing to meet the body’s metabolic needs and contributing to substantial global morbidity and mortality [1]. The recent epidemiological studies indicate that heart failure affects 64.3 million individuals globally, with the highest burden observed in the aging population. Notably, ischemic heart disease remains the leading cause of cardiac-related deaths, accounting for approximately 7.2 million fatalities annually [2]. HF primarily develops from two distinct groups, including dilated cardiomyopathy (DCM) and Ischemic cardiomyopathy (ICM) [3]. DCM is characterized by left ventricular dilation, impaired systolic function, and reduced ejection fraction [4], while genetic mutations in sarcomeric and cytoskeletal proteins play a central role in its etiology, Secondary contributors include viral myocarditis, toxic exposures (e.g., alcohol or chemotherapy drugs), and metabolic disorders, all of which contribute to disease pathogenesis [5]. Ischemic Cardiomyopathy (ICM), primarily resulting from coronary artery disease, through myocardial ischemia, cell death, fibrosis, and left ventricular remodeling, ultimately reduces cardiac output. Like DCM, with which it shares pathological mechanisms, it includes oxidative stress, neurohormonal dysregulation, and mitochondrial dysfunction [6,7].

In the past three decades, pharmacological management of heart failure has been revolutionized by renin–angiotensin–aldosterone system (RAAS) inhibitors and β-adrenergic antagonists, leading to substantial improvements in outcomes [8]. Nevertheless, these treatments exhibit significant limitations, including the dose-limiting adverse side and incomplete long-term therapeutic efficacy. These constraints highlight a critical unmet need for novel treatment modalities that can more effectively restore cardiac function and prevent disease progression. Innovative therapeutic strategies enhance heart function and prevent disease progression [9,10,11].

Traditional Chinese Medicine (TCM) has emerged as a promising complementary therapeutic strategy for HF, owing to its inherent polypharmacological properties that enable modulation of multiple pathological pathways [12,13,14,15]. Bioactive constituents within established herbal formulation [16] demonstrate target regulation of key molecular mechanisms involved in HF pathogenesis. However, despite growing scientific interest, comprehensive system-level analysis of herbal medicine mechanisms in HF remains critically underexplored, representing a significant gap in the current approach. Systemic investigations elucidating the mechanistic pathways of herbal therapeutic in heart failure remain limited.

To address this gap, our study utilizes a network pharmacology approach to explore the molecular mechanisms and pharmacodynamic interactions of herbal compounds in HF [17,18]. By integrating computational predictions with experimental validation, our aim is to use herbal medicine research and provide novel insights into alternative therapeutic strategies for HF treatment.

## 2. Result

### 2.1. Active Compounds in Herbs and Targets

**Figure 1:**
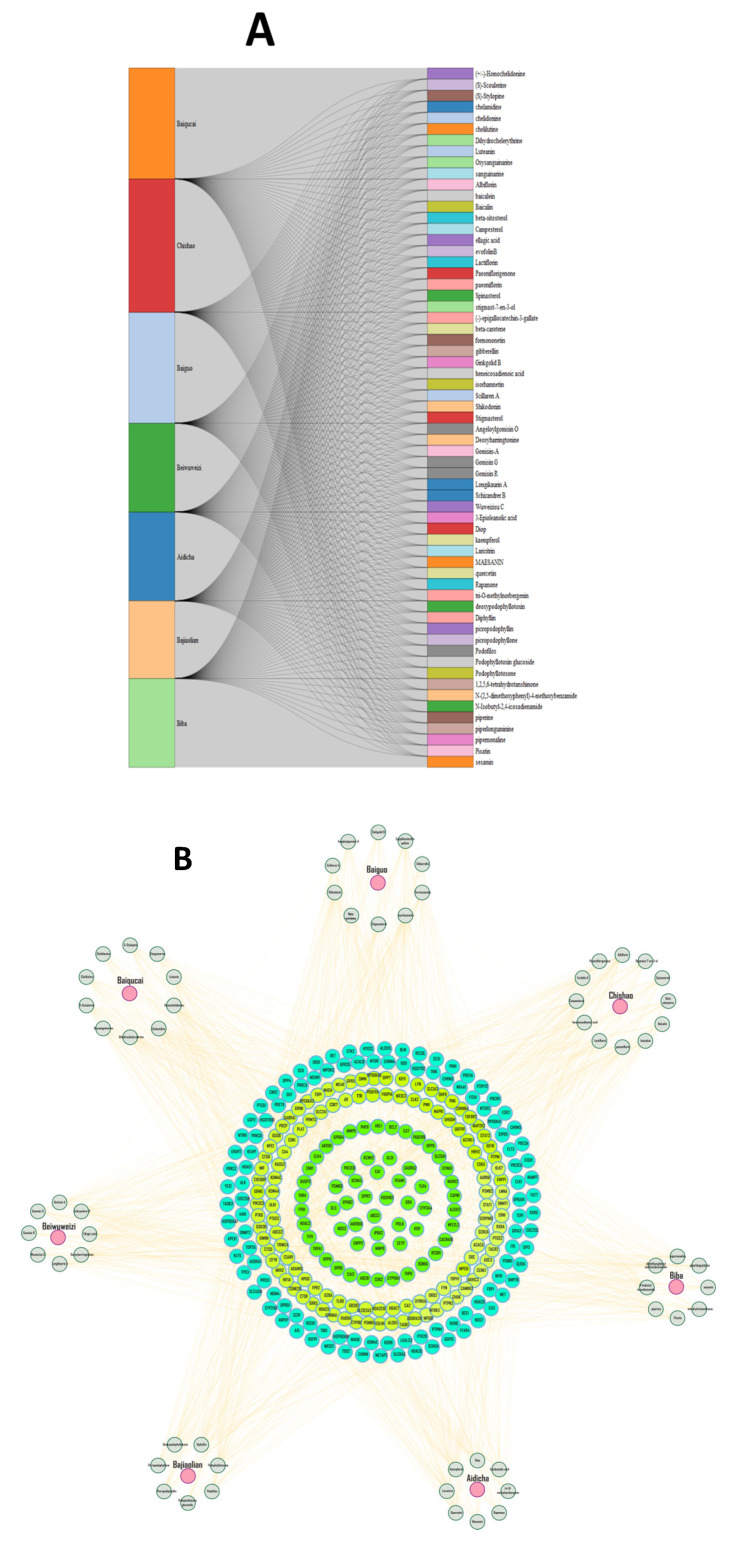
Screening of herb active compounds and targets in HF. (**A**) Sankey diagram of associations between herbs and their associated Chinese herbal compounds. (**B**) Demonstrates the herb–compound target network.

A total of 63 chemical compounds were retrieved from the TCMSP database (Figure 1A, Supplementary Table S1), and a potential 1947 target of the identified compounds were predicted using the SuperPred database (Supplementary Table S2). The herb–compound target network was constructed in Cystoscope, comprising 351 nodes and 1981 edges (Figure 1B). A network analysis revealed that quercetin (degree:48), epigallocatechin 3 gallate (degree:39), and kaempferol (degree:39) were key nodes. Genes associated with heart failure were identified and collected from three distinct databases: GeneCards, OMIM, and TTD. A total of 16,324 non-redundant genes were compiled (Supplementary Table S3). To further refine the dataset, a Venn analysis was performed, identifying 265 shared genes between HF and the herb-derived targets.

### 2.2. Differential Expression Genes in Heart Failure

**Figure 2:**
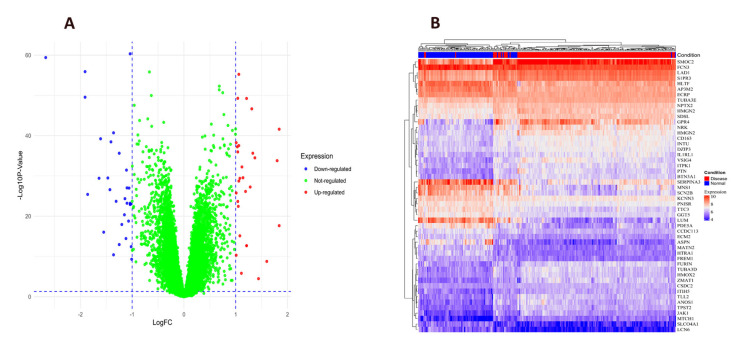
Herb evaluation to identify common targets for HF. (**A**) The differential gene volcano map depicts the gene expression in disease specimens. Green denotes no discernible change, whereas red and blue indicate upregulated and downregulated genes, respectively. (**B**) The gene expression levels for all 48 DEGs are shown in heatmaps. Rows represent the genes, while columns represent the samples.

Differentially expressed genes (DEGs) associated with heart failure HF pathogenesis were identified (Supplementary Table S3). A volcano plot and heatmap were generated to visualized the top genes, which included 22 upregulated genes and 26 down-regulated genes (Figure 2A,B, Supplementary Table S4). The top gene were also summarized in (Table 1). Several identified genes have been implicated in HF pathophysiology. The Secreted modular calcium-binding protein 2 (SMOC2), a cysteine-rich acidic secreted protein, has been reported to link to cardiac diseases progression [19]. The high expression of *FREM1* and *MNS1* suggests their involvement in bile acid, fatty acid, and heme metabolism, potentially contributing to HF progression. Additionally, FCN3 and SERPINA3 are associated with xenobiotic metabolism, inflammatory response, and adipogenesis [20]. Downregulation of TUBA3D and TUBA3E has been identified in dilated cardiomyopathy (DCM,) and may be a significant molecular alteration contributing to the disease [21].

Several genes have been proposed as potential biomarkers for HF diagnosis. *FREM1* has been identified as a diagnostic marker for HF [22], while LAD1 has also been suggested as a candidate diagnostic biomarker in HF [23]. The sphingosine-1-phosphate receptor 1 (S1PR1), a G-protein-coupled receptor, has been implicated in HF pathogenesis [24]. Additionally, the differential expression of HLTF and AP3M2 compared to controls suggests their potential roles in HF progression [25,26,27]. These findings provide crucial insights into the molecular mechanisms underlying HF and lay the foundation for future research in drug discovery, therapeutic development, and experimental validation.

### 2.3. Construction of PPI Network for Common Targets

**Figure 3:**
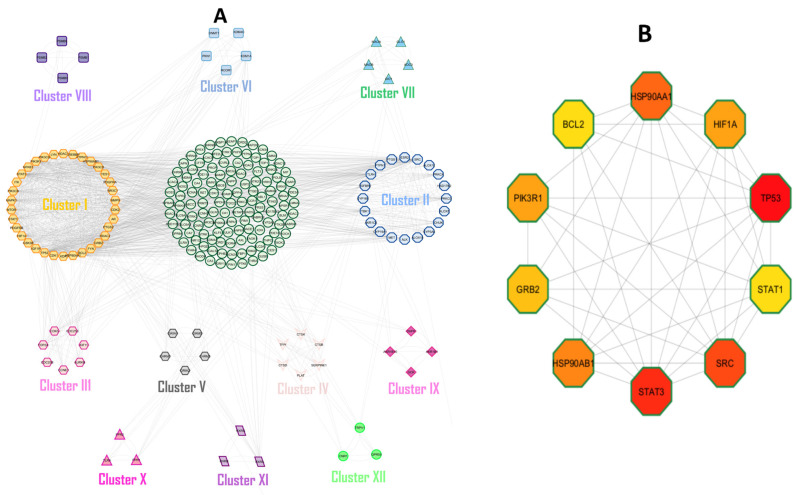
Protein–Protein Interaction (PPI) analysis of HF-related targets. (**A**) Representation of the PPI network and 12 interconnected clusters derived from the network. (**B**) Identification of top core targets based on DC ≥ 2 times the median DC.

STRING and Cystoscope were used to establish the Protein–Protein Interaction network encompassing 265 common targets. The network includes 225 nodes and 1578 edges, divided into 12 distinct clusters (Figure 3A). the cyto-Hubba plugin employed for topological analysis, 10 core targets were screened out based on the degree of centrality median (Table 2, Figure 3B). Among these, the top three proteins Signal Transducer and Activator of Transcription 3 (STAT3), Tumor Protein P53 (TP53), and Proto-oncogene Tyrosine-Protein Kinase Src (SRC) were selected for molecular docking and simulation analyses due to their high degree of centrality and well-established roles in heart failure (HF).

### 2.4. Evaluation of mRNA Levels and Target-Organ Analysis

**Figure 4:**
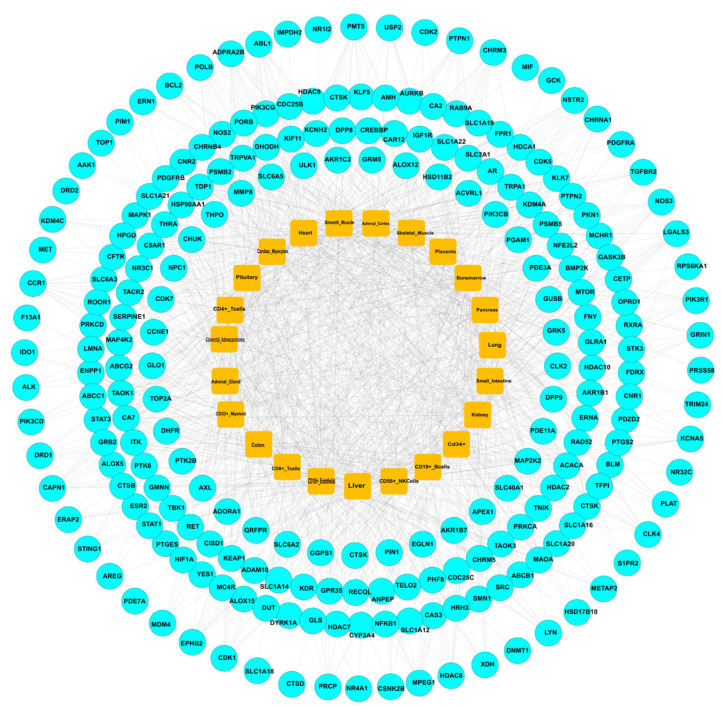
The target-organ network analysis; the top 23 organs have been selected based on highest frequency in the network.

The systemic pharmacological effects of herbal treatment for HF were evaluated by analyzing the mRNA expression profiles of 265 PPI targets using the BioGPS database. The target-organ network created by Cytoscape 3.9.1 revealed associations with 23 organs, which were divided into three major categories, including immune-related organs, metabolic/hormonal, and other crucial organs (Figure 4). Among immune-related tissues, lymphoid components showed the highest degree of involvement. CD4 + T lymphocytes displayed the greatest number of target expressions (1167 targets), followed by CD8 + T lymphocytes (126 targets), CD56 + NK cells (70 targets), CD19 + B cells (60 targets), CD105 + endothelial cells (85 targets), and bone marrow (91 targets). These findings highlight the central role of systemic immunity in HF pathology and potential herbal interventions. In terms of metabolic and endocrine systems, key target-rich organs included the adrenals (96 targets), pancreas (46 targets), kidney (65 targets), and small intestine (31 targets), suggesting a regulatory effect on internal homeostasis. Other significant tissues potentially involved in herbal treatment of HF included the heart (72 potentials), smooth muscle (58 potentials), skeletal muscle (37 potentials), placenta (17 potentials), liver (58 potentials), lung (47 potentials), colon (48 potentials), pituitary gland (27 potentials), adrenal gland (65 potentials), colorectal adenocarcinoma tissue (42 potentials), CD 33 + myeloid cells (60 potentials) and CD34 + stem cells (33 targets). Overall, this integrated organ–tissue network emphasizes the systemic pharmacological landscape of herbal medicine in HF treatment, involving immunomodulatory, metabolic, and connective-tissue actions, and supports the concept of multi-target therapeutic action.

### 2.5. GO Enrichment Analysis

**Figure 5:**
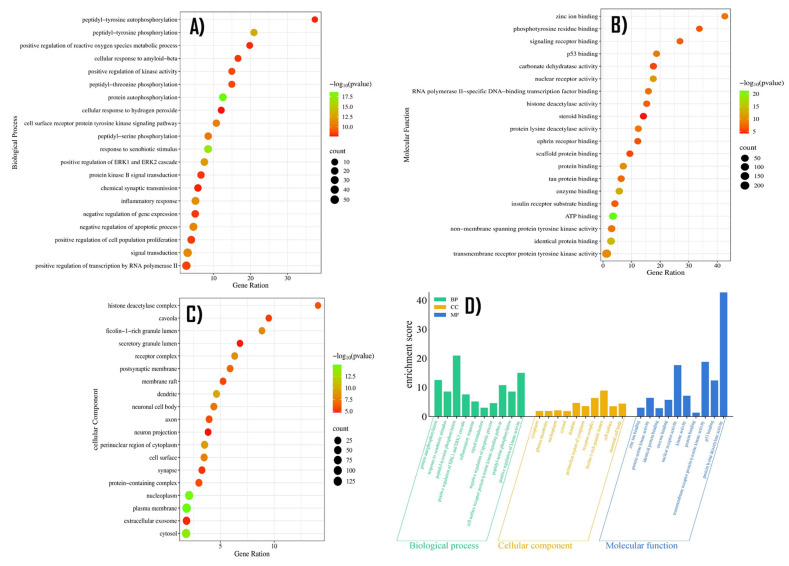
Results of Go and keg enrichment analysis are illustrated in the above figure. (**A**) Bubble plot of the top 20 biological process (BP) terms involved in the GO enrichment analysis. (**B**) Bubble plot exploring GO cellular component (CC) terms obtained from enrichment analysis. (**C**) Bubble plot of molecular function (MF) terms from Go enrichment analysis. In all plots the horizontal axis is reserved for the gene ratios, while the vertical axis denotes the detail of the process. The color of each bubble shows the *p*-value, while the size represents the gene count drawn from micro array. The subsequent bubble plot is constructed in the same manner as described above. (**D**) exhibits the bar plot of the 10 most enriched GO terms in BP, CC and MF groups, represented as green, orange and blue bars, respectively.

Gene Ontology (GO) was conducted on the identified targets using thresholds of *p* < 0.05 and FDR < 0.05. The results yielded a total of 569 Biological process (BP), 103 Cellular components (CCs), and 183 Molecular functions (MFs), as illustrated in Figure 5 and detailed in Supplementary Tables S5–S7. Among the BP terms, the most enriched processes included protein autophosphorylation, peptidyl-tyrosine phosphorylation, positive regulation of kinase activity, inflammatory response, and positive regulation. In the CC terms, the most significant terms were related to Ficolin-1- rich granule lumen, receptor complex, and dendrite raft and cell surface. Regarding MF terms, key functions involved protein lysine deacetylase activity, p53 binding, transmembrane receptor protein, tyrosine kinase activity, and nuclear receptor activity. This analysis highlights the multi-functional roles of the target genes, particularly in signal transduction, immune regulation, and epigenetic modification, which may underlie the therapeutic effects of herbal interventions in HF.

### 2.6. Docking and Evaluation

To validate the results of the network pharmacology study, molecular docking was performed using AutoDock Vina 1_1_2 to analyze the interactions between the selected chemical ligands and key target proteins. The key proteins were prioritized based on network pharmacology analysis, including STAT3 (modeled using the AlphaFold structure P40763), TP53 (crystal structure PDB ID: 7XZZ), and SRC (crystal structure PDB ID: 2BDJ). These targets were subjected to docking simulations to evaluate binding affinities and interaction patterns. The binding sites of each protein were analyzed using grid boxes, according to their structural features [28,29]. We mapped the STAT3 binding pocket by constructing a 46 × 46 × 44 Å grid box (0.375 Å resolution) centered at coordinates (X: 24.946, Y: 13.375, and Z: −27.67 Å) to ensure precise molecular docking. In the case of the SRC binding region, the cubic grid box was employed (40 × 40 × 40 Å), precisely centered at coordinates (X: 10.722, Y: −3.342, and Z: 25.056 Å). For the TP53 protein, we implemented an asymmetric grid box (28 × 36 × 30 Å), with spacing resolution (0.43 Å) strategically positioned at coordinates (X: 99.22, Y: 177.75, and Z: 141.70 Å) to ensure optimal coverage of its binding site. Through a comprehensive literature study, we identified the crucial binding regions of proteins. For the STAT3 protein, the catalytic pocket was characterized by residues (K591, R609, S611, S613, V637, and P639) [30]. In contrast, the SRC active site comprised a distinct set of amino acids including S273, R279, S280, R281, and R283, along with hydrophobic F382 residues, which contributes to its unique binding properties.

To evaluate the pharmacokinetic properties and drug-likeness of the identified bioactive compounds, analyses were conducted using the SwissADME tool. The results, summarized in Table 3, demonstrate that most compounds exhibited favorable oral bioavailability and acceptable pharmacokinetic characteristics, according to Lipinski’s Rule of Five. Specifically, quercetin, kaempferol, and epigallocatechin satisfied the key parameters, including molecular weight, hydrogen bond donors and acceptors, lipophilicity (LogP), and topological polar surface area (TPSA), indicating good oral absorption potential. These findings suggest that the selected compounds possess desirable physicochemical and pharmacokinetic properties, supporting their potential as orally active therapeutic candidates for the treatment of heart failure.

**Figure 6:**
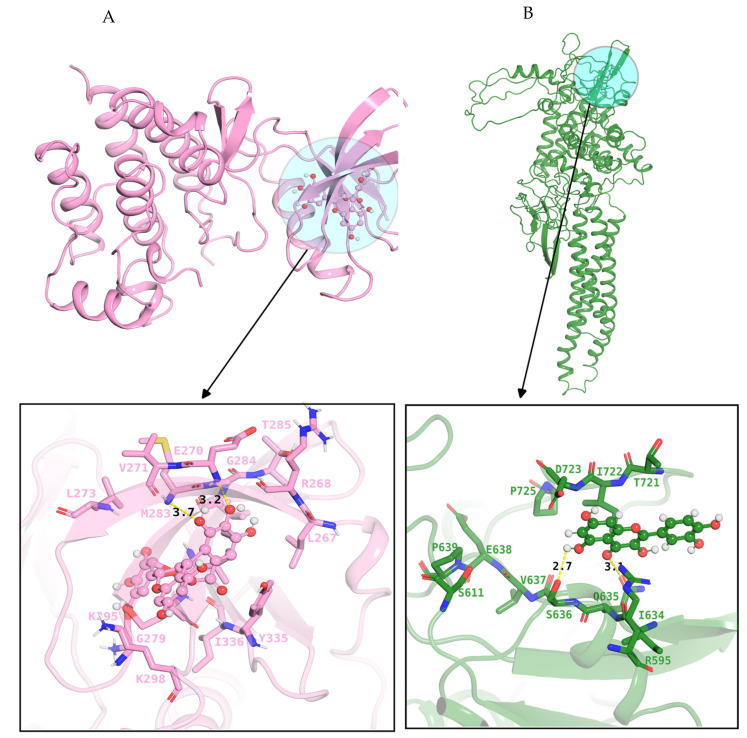

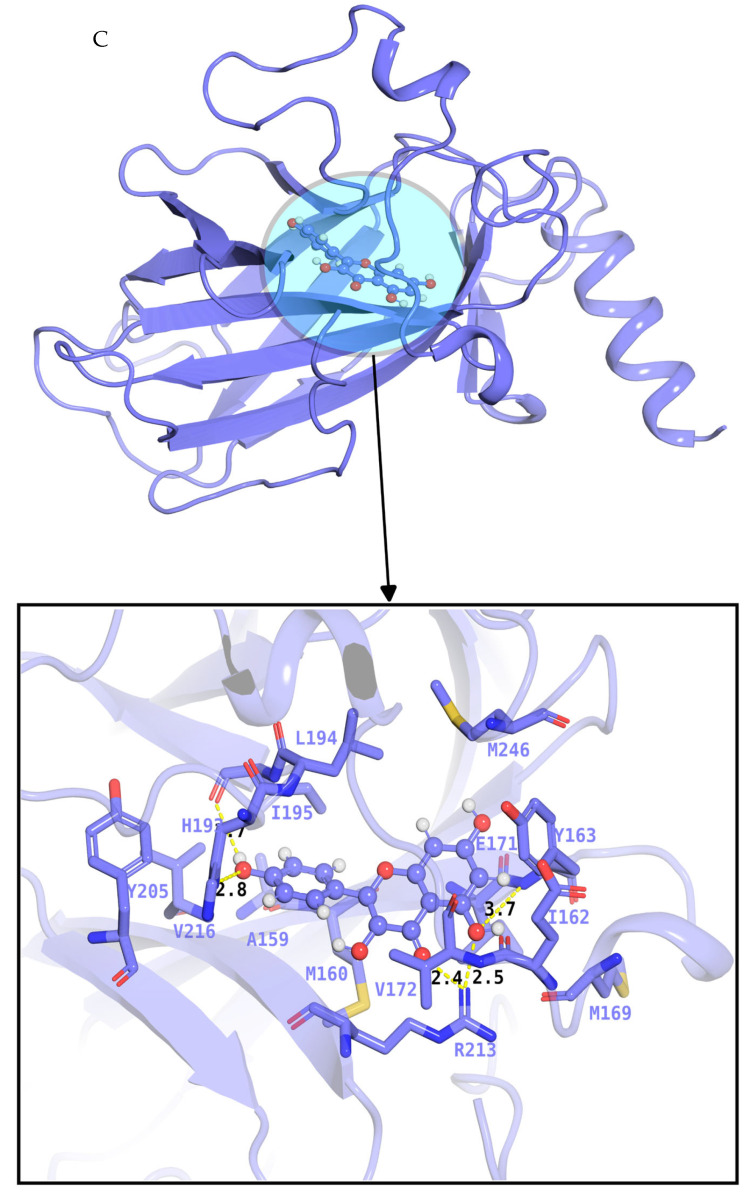
The docking complexes of targeted proteins with best binding compounds. (**A**): (pink color) represents the STAT3–quercetin complex, while (**B**) (green color) demonstrates the binding mechanisms of the SRC–epigallocatechin complex. Similarly, (**C**) (highlighted in blue color) explores the binding modes of the TP5–kaempferol complex.

In this study, molecular docking was performed to validate representative compound-target interactions within the broader multi-target framework of Traditional Chinese Medicine (TCM) [31]. While TCM exerts therapeutic effects through the synergistic modulation of multiple proteins and pathways, selective docking analyses were conducted to illustrate key interactions underlying its pharmacological relevance, rather than to imply single-target specificity. Moreover, potential off-target interactions were considered by integrating target prediction data from resources such as SwissTargetPrediction and SuperPred, ensuring a more systematic assessment of TCM’s polypharmacological behavior [32]. Molecular docking analysis revealed distinct binding profiles between phytochemical compounds and their respective target proteins [33]. The STAT3–quercetin complex demonstrated strong affinity (∆G=−8.3 Kcal/Mol), stabilized by an extensive hydrogen bond network between the ligand’s hydroxyl groups and active site residues R595, S636, and V637 of the protein. Similarly, the binding profile of the TP53–kaempferol complex exhibited strong binding energy (∆G =−7.8 Kcal/Mol), with stability mediated through interactions with key residues R213, H214, I195, H193 and E171. In the case of the SRC-epigallocatechin-3-gallate (EGCG) complex, this exhibits favorable binding energy (ΔG = −7.3 kcal/mol), indicating strong and stable interaction with the protein. Notably, these residues E270, M283, and T296 were involved in the forming of hydrogen bonds and van-der-Waals contact with the hydroxyl group and oxygen atoms of ligands. Hydrogen bonds are illustrated as yellow dashed lines in Figure 6, highlighting the molecular interactions that underlie the binding specificity and strength. These results provide structural support for the potential therapeutic effects of natural compounds on HF-related targets. To validate the binding modes of protein–ligand complexes, they were subjected to molecular dynamic simulation.

### 2.7. Molecular Dynamics Simulation Analysis

**Figure 7:**
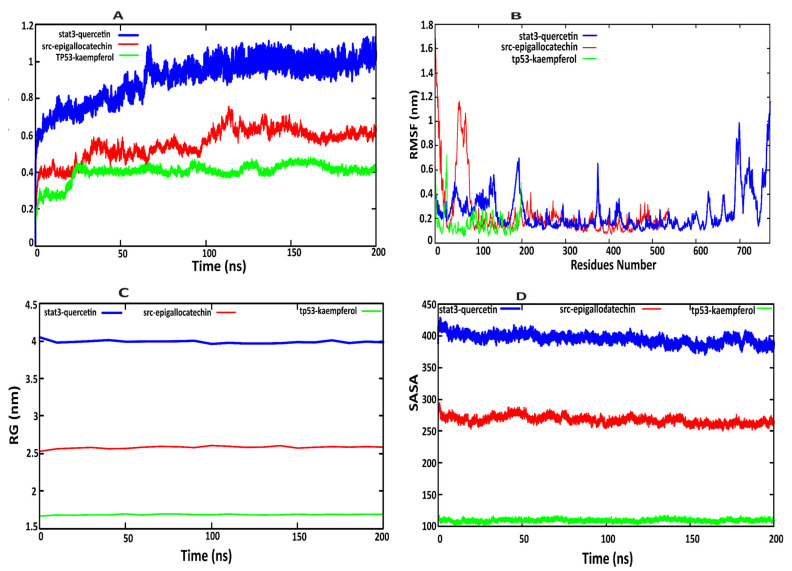
Molecular dynamics simulation (200 ns) analysis of three ligand–protein complexes: (**A**) RMSD plot stat3–quercetin indicated with blue lines, with the RMSD plot for SRC–epigallocatechin (red) and the RMSD plot for tp53–kaempferol (green). (**B**) represents the RMSF plot of three complexes, (**C**) exhibits the radius of gyration and (**D**) demonstrates the radius of SASA of protein– ligand complexes.

Molecular dynamics (MD) simulations of 200 ns were performed using Maestro software to explore the structural dynamics and stability of three protein–ligand complexes: STAT3–quercetin, SRC–epigallocatechin gallate, and TP53–kaempferol. Four key parameters were analyzed—Root Mean Square Deviation (RMSD), Root Mean Square Fluctuation (RMSF), Radius of Gyration (Rg), and Solvent Accessible Surface Area (SASA), to provide insights into the conformational behavior and stability of the complexes. The RMSD plots (Figure 7A) revealed that the STAT3–quercetin complex exhibited significant structural rearrangement, with RMSD values reaching 1.0 nm around 60 ns, suggesting destabilization of the native conformation upon ligand binding. The slight RMSD variation may be attributed to the intrinsic flexibility of the STAT3 protein, particularly within its SH2 domain, which undergoes conformational changes during ligand binding. Such fluctuations are common in dynamic proteins, and can reflect local adjustments that facilitate stable binding rather than global instability. Overall, despite minor structural fluctuations, the docking score and interaction profile support the fact that the STAT3–quercetin complex remains stable and biologically relevant. The stepwise changes in the trajectory indicate potential conformational shifts affecting biological activity. In contrast, the SRC–epigallocatechin complex maintained low and stable RMSD values (~0.2−0.4 nm), indicating strong interactions and minimal structural deviation. The TP53–kaempferol complex showed moderate stability, with RMSD peaking at 0.4 nm within 20 ns and remaining steady afterward, reflecting flexible and stable dynamics.

RMSF analysis (Figure 7B) assessed residue-level flexibility in the protein–ligand complexes. The STAT3–quercetin complex exhibited high fluctuations in several loop regions, particularly residues 40–50, 125–140, 180–200, 185–196, 370–390, 380–400, 681–683, 690–700, and 740–770, which may facilitate ligand adaptability. These elevated fluctuations are primarily due to the presence of the C-terminal SH2 domain and flexible loop regions, which are intrinsically disordered. Despite these fluctuations, the active site of STAT3 demonstrated stability in several regions, as indicated by the RMSF plot, suggesting that the ligand-binding pocket remains structurally reliable. In contrast, the SRC–epigallocatechin complex initially showed localized flexibility at residues 50–60 but stabilized rapidly, reflecting a compact and stable complex. TP53–kaempferol demonstrated moderate fluctuation around residues 20–25, likely due to loop dynamics, followed by stable behavior throughout the simulation. The (Rg) analysis measured structural compactness. The STAT3–quercetin complex exhibited the highest Rg values, correlating with its increased RMSD and structural expansion. In contrast, the SRC–epigallocatechin complex maintained a compact structure, with an Rg around 2.5 nm. The TP53–kaempferol complex showed relatively low Rg (~1.6 nm), indicating sustained structural integrity (Figure 7C). The SASA results revealed that the STAT3–quercetin complex showed an increase in solvent exposure, with values ranging from $380\;to\;410\;{\mathrm{nm}}^{2},$ further supporting the notion of conformational destabilization. The SRC–epigallocatechin complex maintained SASA values between $270\;and\;300\;{\mathrm{nm}}^{2},$ suggesting effective shielding of hydrophobic regions and enhanced complex stability. The TP53–kaempferol complex showed SASA values between $100\;and\;110\;{\mathrm{nm}}^{2},$ indicating dynamic controlled solvent interactions (Figure 7D).

Collectively, the MD simulation results suggest that the STAT3–quercetin complex is structurally less stable, whereas the SRC–epigallocatechin complex forms a highly stable and compact interaction. The TP53–kaempferol complex displays intermediate flexibility, potentially facilitating its biological function. These findings contribute to a deeper understanding of the structural basis underlying the stability and therapeutic potential of the investigated protein–ligand complexes, 180–200, 370–390, 681–731, and 740–770. These residues have higher Root Mean Square Fluctuation.

## 3. Discussion

Heart failure (HF) is a multifactorial clinical syndrome characterized by impaired ventricular filling or ejection, leading to structural and functional abnormalities of the heart. Despite advances in pharmacological interventions, including β-blockers, ACE inhibitors, and RAAS inhibitors, current therapies are often limited by adverse effects, reduced long-term efficacy, and an inability to completely reverse cardiac remodeling and hypotension, renal impairment, or fatigue [34]. Consequently, there is growing interest in exploring complementary therapeutic approaches such as Traditional Chinese Medicine (TCM), which has been extensively used for decades in the prevention and treatment of HF, offering promising therapeutic outcomes through multi-target and multi-pathway regulation. These natural compounds, including flavonoids, lignans, and saponins, exert cardioprotective effects through multi-target regulation, such as enhancing antioxidant defense, reducing inflammation, improving mitochondrial function, and attenuating cardiac fibrosis [35]. Clinical evidence has also demonstrated that TCM formulations exert fewer adverse effects and exhibit synergistic efficacy when combined with conventional Western medicine. In the present study, we integrated network pharmacology, molecular docking, and molecular dynamics (MD) simulation to systematically investigate the underlying mechanisms of TCM in the treatment of HF. Active compounds and disease-associated targets were collected from the TCMSP and GeneCards databases. The herb–compound target interaction network was established to identify the bioactive molecules that play pivotal roles in modulating HF-related pathways. Subsequently, a protein–protein interaction (PPI) network was constructed, followed by Gene Ontology (GO) and Kyoto Encyclopedia of Genes and Genomes (KEGG) enrichment analyses to explore the biological functions and signaling pathways associated with the identified targets.

Similarly, Xiankuan Li et al. employed a comparable integrative strategy to explore the bioactive compounds and molecular mechanisms of Schisandrae chinensis Fructus against drug-induced liver injury [36]. Xiaoming Yu analyzed the molecular mechanisms of Xuefuzhuyu decoction in the treatment of pulmonary hypertension using network pharmacology, further validating the findings through molecular docking [37]. Xin Ren adopted a similar approach—combining network pharmacology, molecular docking, and molecular dynamics simulation—to identify potential Chinese medicines for the treatment of Alzheimer’s disease [38]. Ying Wang investigated the therapeutic effects of Qingfeiyin in treating acute lung injury by integrating GEO dataset analysis, network pharmacology, and molecular docking [39]. Likewise, Zexing Chen applied a similar methodology involving network pharmacology, molecular docking, and molecular dynamics simulation to identify novel bioactive compounds from Radix Bupleuri for the treatment of depression associated with SARS-CoV-2 infection [40].

Furthermore, transcriptomic data from the BioGPS database were employed to evaluate the tissue-specific expression of potential targets, thereby enhancing the reliability of the results. During PPI network construction, key proteins such as STAT3, TP53, and SRC were identified, each of which plays a well-documented role in the pathophysiology of heart failure. Literature validation confirmed that the inhibitor protein IKKα binds to STAT3 and promotes phosphorylation at residue S727. Suppression of STAT3-S727 phosphorylation has been shown to mitigate dilated cardiomyopathy (DCM) and reduce cardiomyocyte injury [41]. The transcription factor p53, a well-known regulator of apoptosis, angiogenesis, and cellular stress response, has also been implicated in cardiac remodeling and heart failure. Dysregulation of p53 signaling contributes to the loss of cardiomyocyte viability and adverse ventricular remodeling in both hypertrophic and dilated cardiomyopathies [42,43]. Similarly, SRC, a non-receptor tyrosine kinase, regulates cellular growth, survival, and adhesion. Its overactivation has been associated with maladaptive cardiac hypertrophy via the MAPK/ERK and PI3K/AKT signaling cascades, further exacerbating heart failure progression [44].

The identified bioactive compounds, including quercetin, kaempferol, and epigallocatechin, were found to strongly interact with these core targets. Pharmacokinetic evaluation using SwissADME and ADMETsar confirmed that these compounds possess favorable drug-likeness, oral bioavailability, and absorption properties, suggesting their potential to reach effective plasma concentrations through oral administration of the respective herbs. Molecular docking and MD simulations validated their stable binding affinities and conformational integrity within the active sites of key proteins, thereby strengthening the computational predictions.

Previous studies have emphasized the cardioprotective effects of polyphenolic compounds derived from medicinal plants. Kaempferol, a naturally occurring flavonoid, has been reported to attenuate oxidative stress and inflammation in cardiomyocytes by modulating the Nrf2/HO-1 pathway and inhibiting apoptosis [45]. Quercetin, widely distributed in numerous medicinal herbs, has been demonstrated to improve endothelial function, suppress myocardial fibrosis, and enhance cardiac contractility through the regulation of MAPK and NF-κB signaling [46]. Likewise, epigallocatechin-3-gallate (EGCG), the predominant catechin in *Camellia sinensis*, exhibits anti-inflammatory and antioxidant properties that protect against ischemia-induced myocardial injury [47]. Collectively, these compounds highlight the polypharmacological potential of natural flavonoids in cardiovascular drug discovery and their mechanistic relevance to the targets identified in this study.

Our integrative approach provides a holistic understanding of how these compounds may exert synergistic effects in the treatment of HF. Unlike conventional single-target drugs, TCM compounds exhibit multi-target binding behavior, allowing them to simultaneously regulate diverse molecular pathways associated with oxidative stress, inflammation, apoptosis, and mitochondrial dysfunction—key pathological processes in HF. Such a polypharmacological profile aligns with the complexity of cardiovascular diseases, where multiple signaling cascades contribute to disease onset and progression. In conclusion, this study provides a comprehensive systems-level understanding of how natural compounds such as quercetin, epigallocatechin, and kaempferol can target pivotal signaling molecules, including STAT3, TP53, and SRC, to modulate key pathways involved in heart failure. The integration of network pharmacology with molecular docking and dynamics simulations enhances the reliability of these findings. These results not only elucidate the molecular mechanisms underlying the cardioprotective effects of TCM, but also support the future development of natural compound-based multi-target therapies for the effective management of heart failure.

## 4. Materials and Methods

### 4.1. Evaluation of Bioactive Compounds and Associated Molecular Targets

The bioactive compounds were identified from seven medicinal herbs: Baiguo (*Ginkgo biloba*), Chishao (*Radix Paeoniae Rubra*), Biba (*Piper longum*), Aidicha (*Ilex latifolia*), Bajiaolian (*Dysosma* spp.), Beiwuweizi (*Schisandra chinensis*), and Baiqucai (*Sedum sarmentosum*), and used heart failure as a query. The active constituents of these herbs were retrieved from the Traditional Chinese Medicine Systems Pharmacology (TCMSP) (https://tcmsp-e.com/tcmsp.php) database, a comprehensive platform that integrates pharmacokinetic properties and molecular information of traditional Chinese medicines. To ensure pharmacological relevance, compounds were filtered based on Oral Bioavailability (OB ≥ 30%) and Drug-Likeness (DL ≥ 0.18) criteria, allowing the selection of bioactive molecules with favorable absorption and drug-like characteristics for subsequent analysis [48]. A similar methodological approach has been adopted in previous studies, where disease-related keywords (e.g., “rheumatoid arthritis”) were used as search terms in the TCMSP database to systematically identify herbs and their associated bioactive compounds relevant to the disease of interest. This strategy ensures the comprehensive retrieval of pharmacologically active constituents potentially involved in disease modulation [49,50]. Herb–compound relationships were visualized using a Sankey diagram generated with the ggalluvial R package 4.4.1. Compound target genes were retrieved from the TCMSP database and shared targets between herbal compounds and HF-associated genes were identified through a comparative analysis using VENNY 2.1 (https://bioinfogp.cnb.csic.es/tools/venny/index.html, accessed on 7 October 2024) [51].

### 4.2. Integration of Target Gene Identification Methods

HF-related target genes were collected from three databases: Online Mendelian Inheritance in OMIM (https://omim.org/) [52], Gene Cards (https://www.genecards.org/) [53], and TTD (http://db.idrblab.net/ttd/ [54], and redundant entries were removed, and the consolidated target list was analyzed. The Gene Expression Omnibus (GEO; www.ncbi.nlm.nih.gov/geo/) database was searched to identify mRNA expression profiles related to heart failure, and the dataset GSE57338 was selected for detailed analysis. This dataset comprises expression data from 313 individuals, including both heart failure patients and non-heart failure controls [55]. Data processing was performed using the limma packages in R for multi-chip normalization and batch effect correction. Differential gene expression was determined using thresholds of |logFC| > 1 and adjusted *p*-value < 0.05 [56]. Swiss TargetPrediction selects targets with parameter Probability ≥ 0.6 in prediction results for further analysis. The compound’s related targets were identified using the SuperPred website (https://www.hsls.pitt.edu/obrc/index.php?page=URL1216395659, (accessed on 4 October 2025)), and the attribute was set to “homo sapiens”, to predict the targets of the compounds. Out of 63 compounds, we have collected the 1947 potential molecular target of herbal compounds based on the structural similarity using the SuperPred database. To ensure reliability, we applied the standard prediction confidence score (probability ≥ 0.6) and considered only those targets with strong structural similarity to known ligands in the database. This database can predict the potential targets of unknown molecules by calculating the Tanimoto similarity between molecules and more than 300,000 known compounds in the server [57]. Visualization was achieved through ggplot2 (volcano plots) and pheatmap (heatmaps) packages in R. Additionally, HF-associated disease targets were independently acquired from three reference databases [58].

### 4.3. Protein–Protein Interaction (PPI) Network Construction

The shared target genes between the drug and the HF were analyzed using the STRING database (STRING: http://string-db.org). The analysis was constrained to *Homo sapiens* protein interaction, with a stringent confidence threshold set at 0.9 to ensure biological relevance [59]. A PPI network was constructed using Cytoscape software (version 3.9.1), and hub genes were identified using the CytoHubba plugin. Network topology analysis employed three parameters: Betweenness Centrality (BC), Closeness Centrality (CC), and Degree Centrality (DC). The core protein–protein interaction (PPI) network was extracted based on the median centrality value. Additionally, MCODE was employed to performed cluster analysis, revealing a highly interconnected subnetwork of target proteins categorized into 12 groups, as shown in Figure 3B [60,61].

### 4.4. Biological Functional Enrichment Analysis

The drug disease targets were used for Gene Ontology (GO) and Kyoto Encyclopedia of Genes and Genomes (KEGG) enrichment analysis using David (https://davidbioinformatics.nih.gov/geneSearch.html) and SR plot (https://www.bioinformatics.com.cn/srplot, accessed on 8 September 2024). The significance thresholds values were set at *p* < 0.05 and FDR < 0.05. The enrichment results were visualized using bar and bubble plots, where pathway significance was indicated by color gradients (based on −log10 p−values), and gene counts were represented by dot size [62].

### 4.5. Molecular Docking

Molecular docking was performed with three key targets, including STAT3 (AlphaFold model: P40763), TP53 (PDB ID: 7xzz), and SRC (PDB ID: 2bdj) using AutoDock Vina 1_1_2. The protein’s structures were prepared by removing water molecules and ions, adding polar hydrogens, merging nonpolar hydrogens, and defining rotatable bonds. The docking grid boxes were set at known active sites residues of each protein structure to ensure complete binding pocket. Additionally, the three-dimensional structures of compounds were retrieved from PubChem database in SDF format, and converted to a PDB file, followed by energy minimization, using Avogadro software. The prepared ligands were subsequently processed in AutoDockTools 1.5.7 and saved as PDBQT files for molecular docking [63]. Binding modes were visualized using PyMOL 3.1.0 [64]. In this study, the pharmacokinetic profiles of the identified compounds were evaluated using computational tools, including SwissADME, to assess Lipinski’s Rule of Five and related pharmacokinetic parameters. Among the screened compounds, quercetin, kaempferol, and epigallocatechin satisfied these criteria, indicating favorable drug-likeness and absorption properties. Therefore, these compounds were selected for further analysis [65].

### 4.6. Molecular Dynamics (MD) Simulation

Molecular dynamics (MD) simulations were performed to evaluate the structural stability of the protein–ligand complexes (STAT3-quercetin, TP53-kaempferol, and SRC-epigallocatechin) using Desmond v4.3 software (Schrödinger LLC, 2017-4) [66]. Each system was simulated for 200 nanoseconds (ns) with comprehensive analysis of four key parameters: Root Mean Square Deviation (RMSD), Root Mean Square Fluctuation (RMSF), radius of gyration (Rg), and solvent accessible surface area (SASA). These metrics collectively assessed conformational stability, residue flexibility, structural compactness, and surface accessibility, throughout the simulation trajectory. For the 200 ns molecular dynamics (MD) simulations performed in Maestro/Desmond, the OPLS4 force field was employed for both protein and ligand parameterization. Ligand parameters were generated using the LigPrep module in Maestro, followed by force field assignment and charge calculation with the OPLS4 parameter set. The ligand topology and parameters were automatically validated through Desmond’s System Builder, which checks for missing parameters, bond types, and atom typing consistency before simulation. All systems were solvated in an orthorhombic TIP3P water box with appropriate buffer distances, and counterions (Na^+^/Cl^−^) were added to neutralize the system. Energy minimization and equilibration were performed according to Desmond’s default relaxation protocol prior to the 200 ns production run. The use of OPLS4 ensures accurate representation of protein–ligand interactions, and the automated validation in Desmond minimizes the risk of parameterization errors, thus ensuring the reliability of the MD results [67,68,69,70,71].

## 5. Conclusions

This research highlighted an innovative strategy for investigating herbal medicines as potential alternatives in the treatment of heart failure. These herbs included *Baiguo* (*Ginkgo biloba*), *Chishao* (*Radix Paeoniae Rubra*), *Biba* (*Piper longum*), *Aidicha* (*Ilex latifolia*), *Bajiaolian* (*Dysosma* spp.), *Beiwuweizi* (*Schisandra chinensis*), and *Baiqucai* (*Sedum sarmentosum*). Their mechanisms of action in heart failure were explored using the herb–compound target network. A total of 265 target genes were identified by intersecting compound-related targets with HF-related genes. These targets were used to construct a protein–protein interaction network and the target-organ association network. Gene ontology and KEGG enrichment analysis revealed that these targets participate in key biological process and pathways relevant to the therapeutic effects of these herbs. Notably, STAT3, TP53, and SRC emerged as central nodes in the networks. Molecular docking and molecular dynamics simulation validated the compound target interaction. Over the course of the simulation study, the STAT3–quercetin complex displayed considerable dynamic instability. This was a key finding from the trajectory analysis, where the complex’s high RMSD profile served as a primary metric, pointing to significant fluctuations and large-scale conformational changes within the complex’s structure over time. In contrast, the SRC–epigallocatechin and TP53–kaempferol complexes showed moderate RMSD and radius of gyration, indicating more stable binding. These findings suggest that epigallocatechin and kaempferol may serve as therapeutic agent for heart failure. Generally, this study highlights the potential of natural compounds in lead compound discovery for cardiovascular drug development. Furthermore, it bridges the gap between traditional medicine and modern pharmacological science offering a solid foundation for future experimental validation and the development of novel multi-target treatment strategies for heart failure.

## Figures and Tables

**Table 1:** Differential gene information analyzed by GEO datasets.

Gene Symbol	Protein Name	logFC	t	*p* Value	adj. *p* Val	B	Change
TUBA3E	Tubulin Alpha 3E	−1.04	−21.8	4.68 × 10^−61^	1.56 × 10^−56^	128.0436	DR
SERPINA3	Alpha-1-antichymotrypsin	−2.67	−21.5	3.97 × 10^−60^	6.61 × 10^−56^	125.9362	DR
FCN3	Ficolin 3	−1.91	−20.5	1.26 × 10^−56^	1.24 × 10^−52^	117.9906	DR
FREM1	FRAS1 Related Extracellular Matrix protein 1	1.06	20.3	5.86 × 10^−56^	3.90 × 10^−52^	116.4756	UR
HMGN2	Non-histone chromosomal protein HMG-17	0.683	19.4	5.01 × 10^−53^	2.78 × 10^−49^	109.818	UR
ZMAT1	Zinc finger matrin-type protein 1	0.749	19	2.13 × 10^−51^	8.85 × 10^−48^	106.1184	DR
FURIN	Furin	−0.63	−18.8	1.07 × 10^−50^	3.96 × 10^−47^	104.524	DR
IL1RL1	Interleukin 1 Receptor Like 1	−1.91	−18.7	2.79 × 10^−50^	9.30 × 10^−47^	103.577	DR
MNS1	Meiosis Specific Nuclear structural 1	1.04	18.6	5.28 × 10^−50^	1.59 × 10^−46^	102.9497	UR
SMOC2	SPARC Related Modular Calcium Binding protein 2	1.21	18.6	5.75 × 10^−50^	1.59 × 10^−46^	102.865	UR
LCN6	Epididymal-specific lipocalin-6	−0.959	−18.1	2.88 × 10^−48^	7.38 × 10^−45^	99.0001	DR
LUM	Lumican	1.31	17.8	2.20 × 10^−47^	5.23 × 10^−44^	96.99357	UR
KCNN3	Small conductance calcium-activated potassium channel protein 3	0.771	17.4	5.84 × 10^−46^	1.30 × 10^−42^	93.75647	UR
LAD1	Ladinin 1	−0.699	−17.1	6.86 × 10^−45^	1.43 × 10^−41^	91.32271	UR
GGT5	Glutathione hydrolase 5 proenzyme	−0.857	−17	1.68 × 10^−44^	3.29 × 10^−41^	90.43837	DR
MTCH1	Mitochondrial carrier homolog 1	−0.391	−16.9	4.91 × 10^−44^	9.09 × 10^−41^	89.37772	DR
AP3M2	AP-3 complex subunit mu-2	0.529	16.8	1.39 × 10^−43^	2.44 × 10^−40^	88.35005	UR
ITIH5	Inter-alpha-trypsin inhibitor heavy chain H5	0.85	16.7	2.88 × 10^−43^	4.80 × 10^−40^	87.62974	DR
S1PR3	Sphingosine 1-phosphate receptor 3	−0.623	−16.6	4.81 × 10^−43^	7.62 × 10^−40^	87.12467	UR
ECM2	Extracellular matrix protein 2	0.998	16.4	2.19 × 10^−42^	3.31 × 10^−39^	85.62849	DR
ASPN	Asporin	1.84	16.4	2.49 × 10^−42^	3.60 × 10^−39^	85.50125	UR
SLCO4A1	Solute carrier organic anion transporter family member 4A1	0.936	−16.2	1.97 × 10^−41^	2.63 × 10^−38^	83.45463	DR
PDE5A	cGMP-specific 3′,5′-cyclic phosphodiesterase	−1.36	16.1	4.56 × 10^−41^	5.84 × 10^−38^	82.62749	DR
NPTX2	Neuronal pentraxin-2	0.996	−16	6.75 × 10^−41^	8.33 × 10^−38^	82.23937	UR
HLTF	Helicase-like transcription factor	−0.89	16	7.67 × 10^−41^	9.12 × 10^−38^	82.11379	UR
TTC3	E3 ubiquitin-protein ligase TTC3	0.574	15.9	1.20 × 10^−40^	1.38 × 10^−37^	81.66974	DR
PNISR	Arginine/serine-rich protein PNISR	0.406	15.9	2.26 × 10^−40^	2.51 × 10^−37^	81.04558	UR
CD163	Scavenger receptor cysteine-rich type 1 protein M130	0.355	−15.7	5.97 × 10^−40^	6.41 × 10^−37^	80.08657	DR
SDSL	Serine dehydratase-like	−1.61	15.7	6.18 × 10^−40^	6.43 × 10^−37^	80.05264	UR
CSDC2	Cold shock domain-containing protein C2	0.753	−15.6	2.13 × 10^−39^	2.15 × 10^−36^	78.82751	DR
VSIG4	V-set and immunoglobulin domain-containing protein 4	−0.862	−15.5	3.48 × 10^−39^	3.40 × 10^−36^	78.34529	DR
ITPK1	Inositol-tetrakisphosphate 1-kinase	−1.41	−15.5	4.95 × 10^−39^	4.71 × 10^−36^	77.99655	DR
NRK	Nik-related protein kinase	−0.43	15.5	5.49 × 10^−39^	5.08 × 10^−36^	77.89357	UR
ECRP	Eosinophil cationic-related protein	1.01	−15.4	1.17 × 10^−38^	1.05 × 10^−35^	77.14903	UR
TUBA3E	Tubulin alpha-3E chain	−0.909	−15.4	1.28 × 10^−38^	1.12 × 10^−35^	77.05474	UR
MATN2	Matrilin-2	−0.881	15.4	1.36 × 10^−38^	1.16 × 10^−35^	76.99504	DR
ANOS1	Anosmin-1	0.899	15.3	2.04 × 10^−38^	1.70 × 10^−35^	76.59485	DR
DZIP3	E3 ubiquitin-protein ligase DZIP3	0.78	15.3	2.88 × 10^−38^	2.29 × 10^−35^	76.25551	DR
TLL2	Tolloid-like protein 2	0.568	15.3	2.89 × 10^−38^	2.29 × 10^−35^	76.25287	UR
CCDC113	Cilia- and flagella-associated protein 263	1.06	15.3	3.20 × 10^−38^	2.48 × 10^−35^	76.15123	DR
TPST2	Protein-tyrosine sulfotransferase 2	0.781	−15.2	4.66 × 10^−38^	3.53 × 10^−35^	75.78051	DR
GPR4	G-protein coupled receptor 4	−0.424	−15.2	5.40 × 10^−38^	3.99 × 10^−35^	75.63523	UR
PTN	Pleiotrophin	−0.727	15.2	5.88 × 10^−38^	4.25 × 10^−35^	75.55116	UR
HTRA1	Serine protease HTRA1	1.03	15.1	8.40 × 10^−38^	5.95 × 10^−35^	75.19841	DR
JAK1	Tyrosine-protein kinase JAK1	0.58	−15.1	9.85 × 10^−38^	6.83 × 10^−35^	75.04061	DR
BTN3A1	Butyrophilin subfamily 3 members A1	−0.309	15.1	1.15 × 10^−37^	7.79 × 10^−35^	74.89022	DR
SCN2B	Sodium channel regulatory subunit beta-2	0.699	15	1.85 × 10^−37^	1.23 × 10^−34^	74.41531	UR
INTU	Protein inturned	0.86	15	2.29 × 10^−37^	1.50 × 10^−34^	74.20487	UR

UR = upregulated, DR = downregulated.

**Table 2:** Detailed information of the top ten core targets.

No	Uniport IDs	Gene Symbol	Protein Name
1	P04637	TP53	Cellular tumor antigen p53
2	P40763	STAT3	Signal transducer and activator of transcription 3
3	P12931	SRC	Proto-oncogene tyrosine kinase
4	P07900	HSP90AA1	Heat shock protein HSP90-alpha
5	P08238	HSP90AB1	Heat shock protein HSP90-beta
6	P27986	PIK3R1	Phosphatidylinositol 3-kinase
7	Q16665	HIF1A	Hypoxia-inducible factor 1-alpha
8	P62993	GRB2	Growth factor receptor bound protein
9	P10415	BCL2	Apoptosis Regulator BcI-2
10	P42224	STAT1	Signal transducer and activator of transcription 1-alpha/beta

**Table 3:** ADME and drug-likeness parameters of the Chinese medicine.

Name	MW(g/mol)	GI	BBB	PGP	BS	nHBA	nHBD	TPSA(Å)	iLOGP	WLOG	nLV
quercetin	302	high	No	no	0.55	7	5	131.36	1.63	1.99	0
kaempferol	286	high	No	no	0.57	6	4	111.13	1.7	2.28	0
epigallocatechin	458	high	No	no	0.63	9	5	146.14	1.87	1.91	0

*MW* molecular weight, *GI* gastrointestinal absorption, *BBB* blood–brain barrier permeant, *Pgp* P-glycoprotein substrate, *nHBD* number of hydrogen bond donor, *BS* Bioavailability Score, nHBA number of hydrogen bond acceptor, *TPSA* topological polar surface area, *WLOGP* water partition coefficient, *nLV* number of Lipinski violation.

## Data Availability

The datasets analyzed in this study are publicly available in the Gene Expression Omnibus (GEO) database under accession number GSE57338. All other data supporting the result of the article are available in the Supplementary Materials.

## Supplementary Materials

The following supporting information can be downloaded at: https://www.mdpi.com/article/10.3390/ph18111648/s1.
